# Ultrasonic-Assisted Rapid Preparation of Sulfonated Polyether Ether Ketone (PEEK) and Its Testing in Adsorption of Cationic Species from Aqueous Solutions

**DOI:** 10.3390/ma15217558

**Published:** 2022-10-27

**Authors:** Laurentiu Baltag, Corneliu Cojocaru, Andra-Cristina Enache, Petrisor Samoila, Valeria Harabagiu

**Affiliations:** “Petru Poni” Institute of Macromolecular Chemistry, 41A Grigore Ghica 7 Voda Alley, 700487 Iasi, Romania

**Keywords:** sulfonated polyether ether ketone, adsorption, Methylene Blue, modeling and optimization, molecular docking

## Abstract

Herein, we report a new approach for the sulfonation of polyether ether ketone (PEEK) following a shorter path of reaction undertaken at 60 °C under ultrasonication. The application of this method enabled the reduction of the reaction time from several hours to less than one hour, achieving a relevant sulfonation degree. The sulfonated-PEEK (SPEEK) was characterized by advanced chemical and physical instrumental methods. According to ^1^H-NMR analysis, the degree of sulfonation of the polymer was equal to 70.3%. Advanced microscopy (SEM) showed that the fabricated SPEEK beads (2–4 mm) were porous inside with a log-normal distribution of pore sizes within the range 1.13–151.44 μm. As an application, the SPEEK polymer was tested for the adsorption of a cationic organic pollutant (Methylene blue, MB) from aqueous solutions. The equilibrium studies (isotherms) disclosed maximum adsorption capacities of 217 mg/g, 119 mg/g, and 68 mg/g at temperatures of 323 K, 313 K, and 300 K, respectively. The thermodynamic calculations indicated an endothermic effect (ΔH_ad_ = +11.81 kJ/mol) of the investigated adsorption process. The maximum removal efficiency of 99.14% was established by process optimization using the design of experiments strategy and data-driven modeling. Additionally, molecular docking simulations were performed to disclose the mechanism of interaction at the molecular level between the adsorbent (SPEEK) and pollutant.

## 1. Introduction

Poly(ether ether ketone) (PEEK) is a semi-crystalline polymer that possesses excellent mechanical, chemical, and thermal properties, which makes it suitable for many industrial applications, including as a substitute for metals [1]. PEEK is an aromatic polyketone that combines an aromatic backbone and ketone groups. Thus, it is one of the most common engineering thermoplastics used in many fields including electrical, automotive, aerospace, medical, composite systems, and other domains [2,3,4,5,6,7,8,9,10]. Due to its high melt and glass transition temperatures (T_m_ = 340 °C and T_g_ = 143 °C, respectively), PEEK has also been employed in structural and insulation applications [10]. However, the hydrophobic character of PEEK limits its use to certain applications where hydrophilic properties of the polymer are required [11,12]. To overcome this issue, the PEEK can be subjected to a sulfonation reaction to obtain a sulfonated poly(ether ether ketone) (SPEEK) with enhanced hydrophilic properties [11,12,13].

In the last decades, SPEEK has attracted great attention as an ionic material for fabricating proton exchange membranes (PEM) for fuel cell applications [13,14,15,16,17,18,19,20]. For instance, Parnian et al. [14] investigated SPEEK membranes with different degrees of sulfonation as a proton exchange membrane in fuel cell applications. They found that the proton conductivity of SPEEK membranes increased with the degree of sulfonation (DS), and the membranes sulfonated in moderate mode (DS ~ 65%) had acceptable mechanical, thermal, and chemical stability [14]. Chuesutham et al. [15] combined SPEEK and sulfonated Y zeolite (S-Y) to prepare new composite membranes that were tested as alternative materials for the direct methanol fuel cell. These authors disclosed that the proton conductivity values of the composite membranes achieved higher values than those of the commercial Nafion membrane [15]. Likewise, recent studies [21,22,23] have demonstrated that SPEEK has a significant potential for use as a polymeric adsorbent to remove persistent organic pollutants (synthetic dyes) from aqueous solutions. In this case, SPEEK acts as a functional polymer with reactive sulfonic groups (−SO_3_^−^) that can contribute to the retention of organic pollutants by electrostatic interactions (i.e., ion exchange). Kanmaz et al. [23] tested SPEEK for the adsorption of rhodamine B (Rh B) and murexide (MX) dyes from wastewater. They found that SPEEK demonstrated a considerable adsorption ability driven by sulfonic group active sites [23].

Generally, the sulfonation of PEEK can be done at 25–75 °C using concentrated sulphuric acid (96–98%), or a mixture of sulphuric acid with other chemical reagents [11,12]. The activation energy of the sulfonation reaction of PEEK was reported to be around 82.8 kJ/mol [12]. In the PEEK repeating unit, the sulfonation reaction occurs mainly on the phenyl ring flanked by two ether groups [11]. The target extent of sulfonation (30–100%) can be achieved by adjusting different operating parameters, such as acid concentration, reaction time, and temperature. However, it remains of great interest to study the intensification of the sulfonation process by proposing innovative elements.

The main objective of this study is the enhancement of the PEEK sulfonation process by using for the first time an ultrasonic-assisted technique. The use of ultrasound is designed to reduce the reaction time of sulfonation by induction of additional energy into the system. The as-prepared SPEEK material was then tested for the adsorption of cationic pollutants from aqueous solutions, revealing a useful efficiency for this application.

## 2. Materials and Methods

### 2.1. Materials

Poly(ether ether ketone) (PEEK) in the form of powder with a mean particle size of 80 μm was purchased from Merck/Sigma-Aldrich (product no GF75065755). Concentrated sulfuric acid (H_2_SO_4_, 98%) purchased from Roth (product no X945.1) was employed in the sulfonation experiments. The cationic dye Methylene Blue (hereafter, MB), purchased from Sigma-Aldrich (product no M9140), was used in the adsorption assays as a persistent organic pollutant. Other chemical reagents of analytical grade (NaOH, HCl—acquired from the Chemical Company, Iasi, Romania) were used as received without further purification.

### 2.2. Preparation of SPEEK by Ultrasonic-Assisted Technique

The sulfonation of PEEK was performed in a 100 mL Erlenmeyer flask containing 40 mL of 98% sulfuric acid, to which 2 g of PEEK was added. The mixture was stirred continuously at room temperature for 1 h to promote solid-liquid contact and to initiate the dissolution of the polymer. Afterward, the mixture was heated at 50 °C with continuous stirring for 90 min to ensure the complete dissolution of the polymer. The resulting polymeric solution was heated to 60 °C and then immediately transferred to an ultrasonic bath (Emmi-12HC/Emag), where it was subjected to sonication for about 35 min whilst maintaining the temperature at 60 °C. Finally, the sonicated polymeric solution was poured dropwise into cold distilled water, where it precipitated as polymeric beads. The obtained sulfonated PEEK (SPEEK) beads 2–4 mm in size were filtered and washed with distilled water to neutral pH. The advantage of the ultrasonic-assisted technique over the conventional method is that the resulting sulfonated polymer (SPEEK with sulfonation degree > 50%) can be obtained much faster (sonication time below 1 h). In our case, the degree of sulfonation, determined by reverse titration, was found to be 67 ± 2%. Details regarding the reverse titration method used for this purpose are given in the Supplementary Materials (Section S1), and the suggestive scheme of ultra-sonic-assisted SPEEK preparation is depicted in Figure S1 (Supplementary Materials).

### 2.3. Characterization Methods

The SPEEK produced by the ultrasonic-assisted technique was characterized morphologically and structurally. The porous morphology and surface were inspected by scanning electron microscopy (SEM) using an ESCM Quanta 200 spectrometer equipped with an Energy Dispersive X-ray (EDX) module (Brno, Czech Republic). The collected SEM images were further analyzed using ImageJ open-source software. Fourier-transform infrared spectra (FTIR) of the polymeric materials were acquired in the mid-infrared region (4000–600 cm^−1^) using a Bruker Vertex 70 FTIR spectrometer (Ettlingen, Germany). In addition, an optical microscope (Conrad USB, Wels, Austria) was used to examine the morphology of the SPEEK polymeric beads. The thermal and mechanical properties of the samples were studied using a Netzsch TG-DSC Model STA 449 F1 thermal analysis system (Selb, Germany).

### 2.4. Adsorption Experiments

In adsorption assays, the concentrations of MB dye were determined by recording the absorbance on a UV-Vis spectrophotometer (Hitachi UV-2910) at 664 nm wavelength. The fabricated polymeric material SPEEK was tested for the removal of MB dye from aqueous solutions by adsorption (in batch operating mode). In this regard, a biaxial shaker (BIOSAN ES-20/60) fitted with a temperature-control system was used to stir (240 rpm) the working solutions (50 mL). Upon completion of the adsorption, the spent adsorbent (SPEEK) was separated from the liquid phase, and the purified solution was analyzed for the residual amount of MB dye. The adsorption capacity of the polymer SPEEK for MB retention was assessed according to Equation (1):

(1) $$q=\frac{\left(C_{0}-C\right)\times V}{m\times 1000}\;,$$

where q is the adsorption capacity (mg/g), C_0_ is the initial concentration of MB dye (mg/L), C is the final concentration of MB (mg/L), V is the volume of working solution (mL), and m is the weight of the SPEEK adsorbent (g). Additionally, the color removal efficiency Y (%) was also ascertained using Equation (2):

(2) $$Y=\left(1-\frac{C}{C_{0}}\right)\times 100,$$

## 3. Results and Discussion

### 3.1. Characteristics of the Produced SPEEK Polymeric Beads

**Figure 1:**
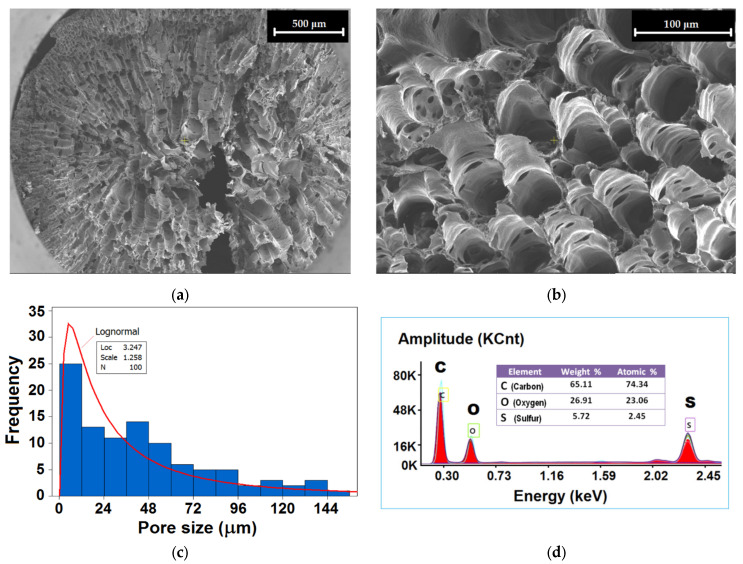
Porous morphology and characteristics of the sulfonated polymer SPEEK: (**a**,**b**) SEM images of the cross-section of a polymeric bead—SPEEK; (**c**) histogram of the pore size distribution; and (**d**) EDX spectrum for the solid surface of the SPEEK adsorbent.

The porous morphology of the produced SPEEK polymeric beads (2–4 mm) was investigated by means of scanning electronic microscopy (SEM). The cross-sectional surfaces of the beads were inspected by SEM and the results are shown in Figure 1a,b. From the SEM images, the size of the pores was recorded and analyzed statistically in order to build the histogram of pore size distribution. According to Figure 1c, the data obeyed the log-normal distribution, and the pore size ranged from 1.13 μm to 151.44 μm, with an average value of 44.31 μm. Based on the location and scale parameters of the log-normal distribution, the mode was found to be 5.28 μm, indicating the most frequent, or probable, pore size in the data set. In addition, EDX analysis (Energy-dispersive X-ray spectroscopy, Figure 1d) revealed the presence of all expected chemical elements (C, O, and S).

**Figure 2:**
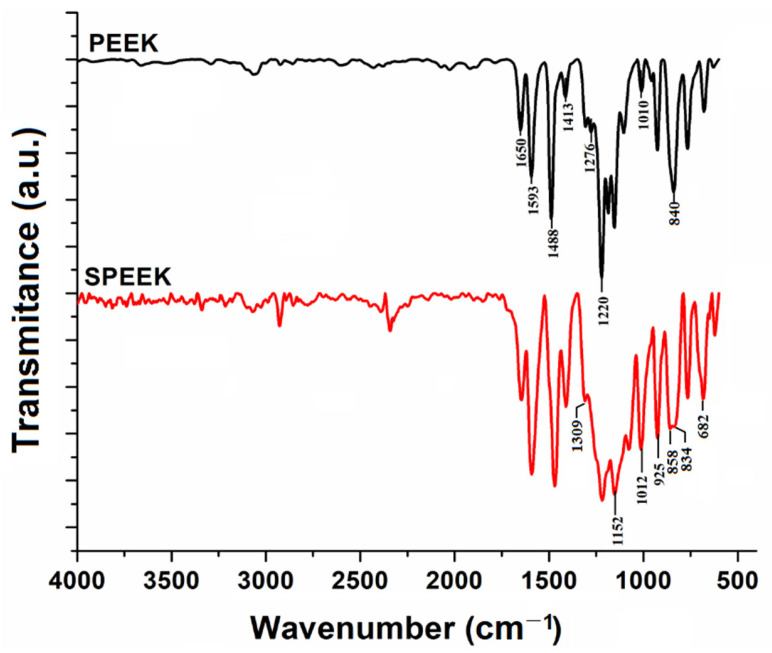
Infrared spectra (FTIR spectroscopy in ATR mode) for pristine polymer (PEEK) sample, and the sulfonated polymer (SPEEK) sample.

Fourier-transform infrared spectroscopy (FTIR) was applied to compare the structural features of the PEEK and SPEEK polymers (Figure 2). Normally, the goal of the infrared analysis is to identify and compare the functional groups of the initial material and its derivatives resulting after chemical modification. The interpretation of infrared spectra was done using available literature information [24]. Figure 2 shows the FTIR spectra of the pristine PEEK polymer and the obtained sulfonated product (SPEEK). The PEEK spectrum shows a carbonyl (C=O) stretching vibration at 1650 cm^−1^, an intense adsorption band at 1220 cm^−1^, a shoulder at 1276 cm^−1^, and a band located at 1010 cm^−1^ suggests the stretching vibration of ether groups (C−O). The presence of the aromatic ring is highlighted by the absorption bands at 1593 cm^−1^, 1488 cm^−1^, and 1413 cm^−1^, which are attributed to the skeletal vibrations, representing (C=C−C) stretching in the aromatic ring [11]. The SPEEK spectrum is similar to that of PEEK as the polymeric backbone chain is the same. However, some modifications appear in the SPEEK spectrum as a result of the sulfonation reaction. Hence, the sulfonic acid groups (−SO_3_H) were evidenced by the =S=O asymmetrical stretching oscillation appearing as a shoulder at 1309 cm^−1^; O=S=O symmetrical stretching vibration pinpointed at 1152 cm^−1^; S=O stretching vibration at 1012 cm^−1^; O−H bending vibration at 925 cm^−1^ and S−O stretching vibration at 682 cm^−1^. Moreover, both PEEK and SPEEK included adsorption bands characteristic to the out-of-plane bending of two hydrogens 1,4-disubstituted phenyl ring (at 840 and 834 cm^−1^, respectively). The sulfonated SPEEK sample revealed a new adsorption band at 858 cm^−1^, which can be attributed to the out-of-plane C−H bending of an isolated hydrogen in the 1,2,4-trisubstituted benzene ring [12]. Likewise, the weak vibrations detectable in the range 3500–3200 cm^−1^ were attributed to O−H stretching from the sulfonic acid groups.

**Figure 3:**
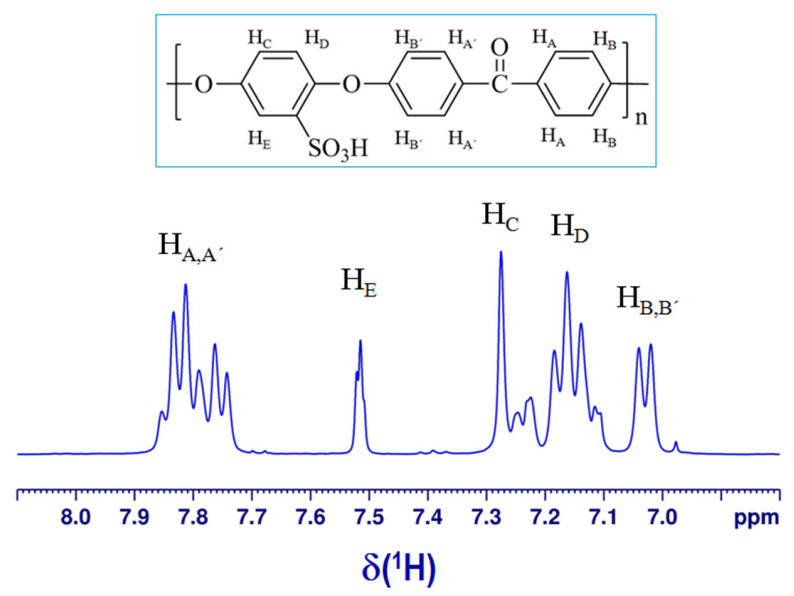
^1^H-NMR spectrum of the SPEEK sample recorded in deuterated solvent (DMSO-d6).

^1^H-NMR spectroscopic analysis was employed to assess the degree of sulfonation (DS%) by a more precise technique. It should be noted that the ^1^H-NMR spectrum for pristine PEEK could not be recorded as this non-sulfonated polymer is insoluble in most organic solvents [12]. Instead, the ^1^H-NMR spectrum of polymeric product SPEEK was recorded in deuterated solvent (DMSO-d6) and is shown in Figure 3. It should be further noted that the ^1^H-NMR signal of the proton from the sulfonic acid group (−SO_3_H) is generally difficult to record due to its labile nature [12]. By contrast, the aromatic proton resonance signals were observed in the ^1^H-NMR spectrum of SPEEK in the interval ranging from 7.00 to 7.85 ppm (Figure 3). It is well known that the sulfonic acid group (−SO_3_H) appears at the phenyl rings between the ether linkages [11,20]. Consequently, the presence of this functional group (−SO_3_H) in the hydroquinone ring of SPEEK is responsible for the downfield shift of the H_E_ proton (7.52 ppm) compared with the other protons from the hydroquinone ring (i.e., H_C_ and H_D_ positioning as doublet/triplet in the range 7.10–7.30 ppm). The other aromatic protons were pinpointed as follows, H_B_ appeared as a doublet at 7.02–7.04 ppm, whereas the multiplet at 7.74–7.85 ppm was attributed to H_A_ protons. Hence, the degree of sulfonation (DS %) of the SPEEK sample can be estimated as the ratio between the area of the H_E_ peak ($A_{H_{E}}$) and the sum of the peak areas ($\sum_{j}A_{H_{j}}$) of the other aromatic protons [11,12,13,20]. To accomplish this, one should first solve Equation (3):

(3) $$\frac{x}{12-2x}=\frac{A_{H_{E}}}{\sum_{j}A_{H_{j}}}$$

where, x is the number of H_E_ per repeat unit (0 ≤ x ≤ 1) that should be established by solving Equation (3); and H_j_ = {H_A_, H_A’_, H_B_, H_B’_, H_C_, H_D_}. In our case, for a ratio of $A_{H_{E}}$/$\sum_{j}A_{H_{j}}$ = 6.636 × 10^−2^, the decision variable was found to be equal to x = 0.7030, which corresponds to a degree of sulfonation of DS = 70.3%. This value for DS (70.3%) determined from the ^1^H-NMR analysis is in reasonable agreement with DS (67 ± 2%) determined by the reverse titration method.

**Figure 4:**
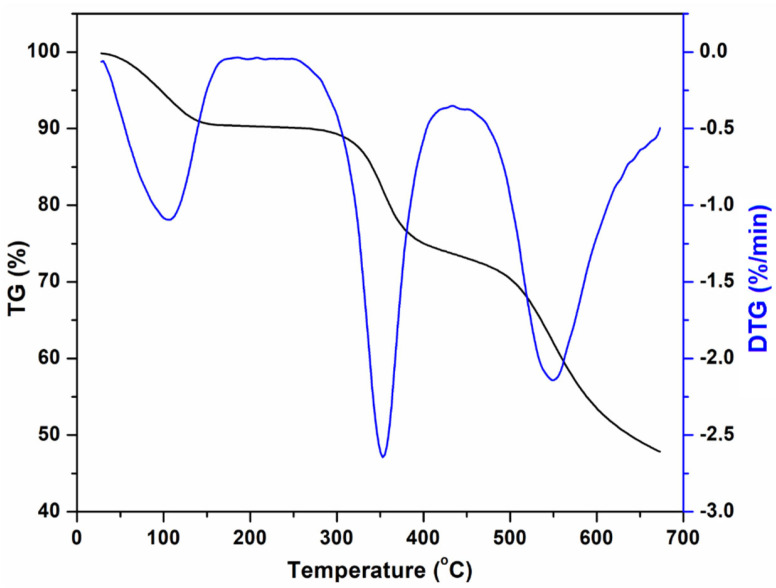
TG and DTG traces showing the influence of temperature on the behavior of the SPEEK polymer (TG—black line, DTG—blue line).

The thermogravimetric (TG) and thermogravimetric derivative (DTG) profiles of SPEEK are shown in Figure 4. As can be seen from Figure 4, three stages of mass loss can be distinguished for the SPEEK sample, that is: (i) 50–200 °C, (ii) 200–430 °C, and (iii) above 430 °C. The mass reduction occurring in the region 50–200 °C is due to the loss of physically and chemically bound water. In the second phase (200–430 °C) the mass loss of the SPEEK sample might be associated with the degradation of the sulfonic acid groups (−SO_3_H), which on cleavage can eliminate SO_3_ by volatilization [12]. And, in the last phase (>430 °C), the mass loss can be attributed to the thermal breakdown of the polymer chain.

### 3.2. Adsorption Assays: Kinetics, Isotherms, and Thermodynamics

**Figure 5:**
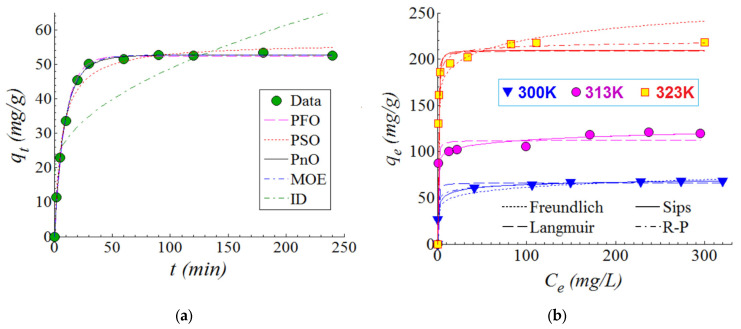
Adsorption of MB cationic dye onto the surface of the SPEEK adsorbent: (**a**) adsorption kinetics (experimental data and mathematical models) at T = 300 K, pH 6.0 ± 0.2, SD = 2 g/L, and C_0_ = 100 mg/L; (**b**) adsorption isotherms (experimental data and mathematical models), conditions: SD = 2 g/L, pH 6.0 ± 0.2, t = 180 min; solid, dashed and dotted lines represent predictions given by mathematical models.

The SPEEK produced by ultrasonic-assisted technique was tested for possible applications by the adsorption of MB cationic dye from aqueous solutions. First, the adsorption kinetics of MB dye onto the surface of the SPEEK polymer were investigated. The kinetics experiments were carried out under the following conditions: T = 300 K (temperature), pH 6.0 ± 0.2, C_0_ = 100 mg/L (initial MB dye concentration), and SD = 2 g/L (sorbent dose). Figure 5a shows the adsorption kinetics results in terms of the dynamics of the adsorption capacity q_t_ (mg/g) versus the contact time t (min). An increase in adsorption capacity generally occurs as contact time increases. According to the results reported in Figure 5a, three stages can be distinguished for the adsorption kinetics. During the first stage, the adsorption capacity increased significantly within the first 20 min. Then, the adsorption capacity increased gradually for the second stage (20–120 min). For the third and final stage (>120 min), the adsorption capacity reached a plateau suggesting the establishment of the equilibrium state (Figure 5a). Experimental data related to adsorption kinetics were interpolated using several kinetic models (PFO, PSO, PnO, MOE, and ID) [25,26,27,28], which are given in Table 1 along with estimated parameters. The kinetic-model parameters were assessed by the non-linear regression technique. As reported in Table 1, q_t_ (mg/g) and q_e_ (mg/g) are the adsorption capacity at any contact time (t) and equilibrium, respectively. The predictions of the kinetic models are shown in Figure 5a as lines with different patterns (solid, dashed, and dotted). The applied kinetic models were evaluated for goodness-of-fit using the chi-squared (χ^2^) statistical test. In general, a small chi-squared (χ^2^) value suggests a better prediction ability of the model. According to Figure 5a and by analyzing χ^2^-values from Table 1, it can be inferred that the best-fitting model of adsorption kinetics was the PnO model (pseudo-n-order) of order n = 1.179, followed by MOE (mix 1,2-order) and PFO (pseudo-first-order) models. Note that, Hizal et al. [21] applied SPEEK polymer, prepared by the conventional method (2 h sulfonation of PEEK), for adsorption of MB dye. These authors showed that the pseudo-second-order kinetic model (PSO) presented the best agreement with the experimental data. In our case, the SPEEK polymer obtained by ultrasonic-assisted technique revealed that the PnO model (with order n = 1.179), which is close to the pseudo-first-order kinetic model (PFO), was a better fit. Thus, it seems that a change in the material preparation procedure can influence its performance, for instance, when it is tested for adsorption kinetics. In addition, the SPEEK polymer revealed a good potential in the adsorption of heavy metal ions (Cd^2+^, Cu^2+^, Pb^2+)^ from aqueous solutions as detailed in Figure S2 and Table S1 (Supplementary Materials).

For the investigated system (SPEEK/MB), the adsorption isotherms were determined at three temperatures (300 K, 313 K, and 323 K). To this end, the contact time was fixed at t = 180 min to attain the equilibrium state of adsorption. The outcomes of the adsorption isotherms are reported in Figure 5b. As one can see from Figure 5b, the greater the equilibrium concentration C_e_ (mg/L), the higher the adsorption capacity q_e_ (mg/g). Moreover, the adsorption of MB dye onto the SPEEK sorbent was favored by the increase in the temperature. In other words, as the temperature increased, the adsorption capacity increased (Figure 5b). The highest adsorption capacity of q_e_ = 217 mg/g was observed at 323 K (50 °C), while adsorption capacities of 119 mg/g and 68 mg/g were observed at 313 K (40 °C) and 300 K (27 °C), respectively (Figure 5b).

Experimental data (the isotherms) were subjected to interpolation using the mathematical models describing the adsorption equilibrium (i.e., Freundlich, Langmuir, Sips, and Redlich-Peterson [29]). The nonlinear regression technique was used to calculate the parameters of the models. The agreement between models and experimental data was also ascertained using the chi-squared (χ^2^) statistical test [29]. The predictions provided by the mathematical models are shown in Figure 5b as lines of different styles (solid, dashed, and dotted). Table 2 summarizes the values of isotherms model parameters as well as the χ^2^-values. According to Table 2, the best-fitting models were found to be the Sips equation, followed by the Redlich-Peterson equation (Table 2).

Additionally, the Dubinin–Radushkevich (D–R) isotherm model [29,30] was employed to identify the type of adsorption process (i.e., physical adsorption, ion exchange, or chemisorption). The principal parameter of the D–R equation that provides information about the nature of the adsorption process is the mean free energy of sorption E_S_ (kJ/mol) [30]. According to this parameter, the adsorption process is predominantly based on physical forces (Van-der-Waals) if E_S_ < 8 (kJ/mol). Conversely, when 8 ≤ ES ≤ 16 (kJ/mol), then the adsorption process relies mainly on ion exchange. If the mean free energy of sorption is even higher E_S_ > 16 (kJ/mol), chemisorption can be the dominating phenomenon [30]. For our investigated system (SPEEK/MB), the mean free energy E_S_ varied from 22.21 to 35.03 (kJ/mol) (Table 2), suggesting that the chemisorption mechanism might be dominant.

**Figure 6:**
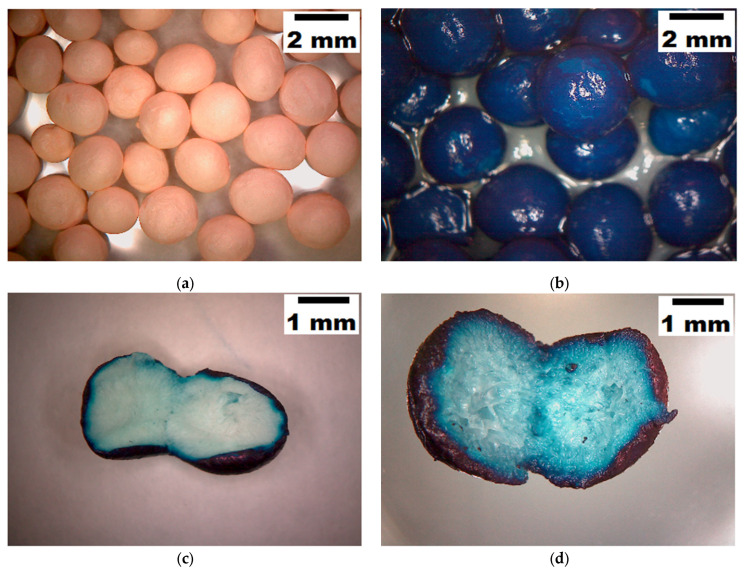
Morphologies of SPEEK beads: (**a**) initial state of SPEEK beads; (**b**) SPEEK beads after MB adsorption; (**c**) cross-section of a SPEEK bead after MB adsorption at 300 K; and (**d**) cross-section of a SPEEK bead after MB adsorption at 323 K.

We also calculated the thermodynamic parameters of the adsorption process for the studied system (SPEEK/MB). More details of the computations of the thermodynamics parameters are given and discussed in the Supplementary Materials (Table S2) [31]. In essence, the calculated negative value of Gibbs free energy of adsorption (ΔG_ad_ = −35.88 kJ/mol) suggested the spontaneous nature of the investigated process. The estimated entropy was found to be positive (ΔS_ad_ = 152.84 J/K.mol), disclosing the accentuating of the state of disorder at the solid-liquid interface. Moreover, the third thermodynamic parameter (enthalpy) was positive (ΔH_ad_ = +11.81 kJ/mol), suggesting an endothermic effect of the adsorption process. According to this endothermic effect, when the temperature is rising, the adsorption capacity increases as well (Figure 5b). In addition, optical microscopy was employed to compare the morphologies of the SPEEK beads in their state (Figure 6a) and after the adsorption of MB dye (Figure 6b). The cross-sections of the SPEEK beads after MB adsorption at different temperatures 300 K (Figure 6c) and 323 K (Figure 6d) were also investigated. According to the microscopic images (Figure 6c,d), as the temperature increased, the diffusion of the MB molecule into the porous matrix of the bead intensified.

### 3.3. Design of Experiments, Multiple Regression Modeling, and Process Optimization

To enhance the performance of the adsorption process using SPEEK beads, we applied the concept of the design of experiments (DoE) that enabled us to study the simultaneous effects of two significant factors over the process response. For this, the response surface methodology (RSM) was employed. More details with respect to the basic concepts of DoE and RSM can be found in the literature [32,33,34]. The chemometric approach involving DoE and RSM allows the study of the synergetic effects of factors on the response in a cost-effective fashion (that is, by reducing the number of required experiments) [32].

The constant conditions for the experimentations involved the following fixed values of operating parameters, sorbent dose SD = 2 g/L; contact time t = 180 min; temperature 323 K; and stirring velocity = 150 rpm (orbital shaker). It should be mentioned here that the temperature was set at 323 K because the isotherms study indicated an enhanced adsorption performance at higher temperatures. As controllable factors (input variables), we investigated the influence of (1) the initial concentration C_0_ of MB dye, and (2) the pH of synthetic wastewater solution. The color removal efficiency, Y (%) was determined as the main response of the adsorption process. Hence, the stated optimization criterion was to find the experimental conditions that maximize the color removal efficiency (i.e., the best response of the adsorption process).

For modeling purposes, the studied factors (C_0_ and pH) were converted into codified input variables x_1_ and x_2_ (varying from −1 to +1) in order to compare their effects within the same dimensionless scale. The expression used for coding factors is reported elsewhere [33,34].

A central composite experimental design (of rotatable type) was employed for carrying out the planned experiments, as summarized in Table 3. In this table, the values of the main factors are reported in actual units (C_0_ and pH) as well as in coded units (x_1_ and x_2_). As given in Table 3, the planned matrix of experimentation involves a total of 11 runs (experimental trials). Each experimental trial (run) represents a unique combination of factors, except for trials 9 to 11, which were explored to estimate the reproducibility of the experiments.

**Figure 7:**
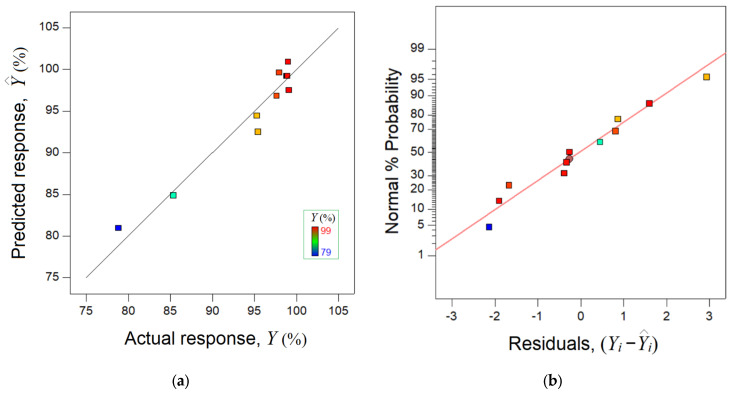
Concordance between experimental data and the predictions given by the model: (**a**) parity plot, and (**b**) normal plot of residuals.

On the basis of the collected data reported in Table 3, a mathematical model was built using the multiple regression technique [33,34]. In terms of coded factors (x_1_ and x_2_), this model can be expressed using Equation (4):

Ŷ = 99.21 − 4.77x_1_ + 1.20x_2_ − 5.76x_1_^2^ + 2.60x_1_x_2_, (4)

subjected to: −1.414 ≤ x_j_ ≤ 1.414; (j = 1,2)

where Ŷ denotes the estimated response (predicted color removal) by the mathematical model. The developed mathematical model (Equation (4)) was subjected to a statistical test in order to prove its adequacy. For this, the analysis of variance (ANOVA) was employed [33,34]. The calculated ANOVA estimators are summarized in Table 4. In accordance with Table 4 (ANOVA), the relevant F-value (26.44) and small *p*-value (0.0006) pointed to a statistically significant model that can be applied to process simulation. The value of determination coefficient R^2^ indicated that the statistical model may explain about 94% of data variation. Moreover, the value of the adjusted coefficient R_adj_^2^ is close to the value of R^2^, suggesting that the mathematical model includes the conformant terms. The mathematical model was diagnosed in terms of the goodness-of-fit (Figure 7). The data located around the bisector (45° straight line) suggests that the predicted values are concordant with the experimental data (Figure 7a). Furthermore, Figure 7b highlights the normal graph of residuals. These represent the difference between observed and predicted values of the response (Y_i_ − Ŷ_i_). Hence, the normal plot of residuals ascertains the deviations of the residual errors from the normal distribution. The larger the deviations from the straight line, the greater the departures from normality. For the studied system (SPEEK/MB), the residual errors were scattered in accordance with the normal distribution (Figure 7b).

By applying the substitution technique, the empirical model with real factors was finally developed as Equation (5):

Ŷ = 98.477 + 0.056 × C_0_ − 1.043 × pH − 2.558 × 10^−4^ × C_0_ ^2^ + 5.773 × 10^−3^ × C_0_ × pH, (5)

subjected to: 38 ≤ C_0_ ≤ 462 (mg/L); 2.8 ≤ pH ≤ 11.2

**Figure 8:**
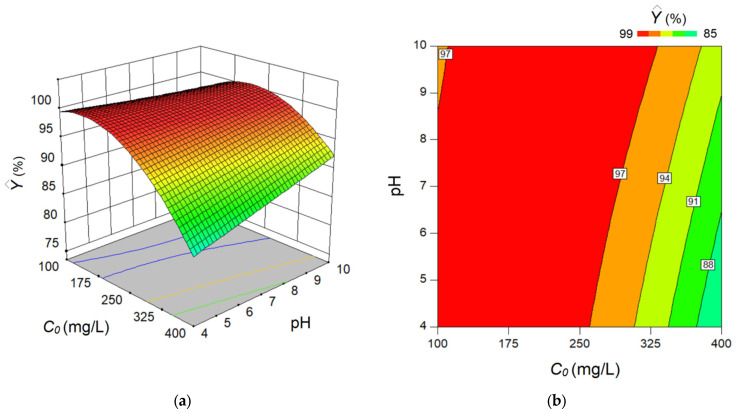
Response surface 3D diagram (**a**) and contour-lines 2D map (**b**) highlighting the mutual effect of factors: C_0_ (initial MB concentration) and pH of the aqueous solution, on the estimated response—removal efficiency (Ŷ, %).

On the basis of this empirical model (Equation (5)), a response surface analysis was performed by plotting the 3D diagram and contour-lines 2D-map in order to disclose the synergetic effect of factors on the estimated response (Figure 8). As shown in Figure 8, the main effect of the C_0_ factor is negative with respect to the estimated response (Ŷ, %). That is, the greater the C_0_ factor, the lower the estimated color removal efficiency (Ŷ, %). According to the response surface analysis, the main effect of the C_0_ factor is more significant than the effect of pH. In addition, an interaction effect exists between these two factors. According to the interaction effect, at higher C_0_ values (>250 mg/L) the increment of pH leads to moderate improvement of the estimated response. By contrast, at low C_0_ values (<120 mg/L) the increase in pH leads to a slight diminishing of the estimated response. For the C_0_ interval of 120–250 mg/L, the changing of pH does not modify the estimated response as a ridge plateau has been attained.

In the last stage, the model-based optimization of the adsorption process was performed. To this end, a numerical optimization was carried out by using the Nelder–Mead simplex method [35]. According to this search algorithm, the optimal values of studied factors were found to be C_0_ = 89 mg/L and pH 2.8. Under these optimal conditions, the maximal color removal efficiency was equal to 99.95% (predicted value—estimated response) and 99.14% (observed value—actual response). The modeling and optimization computations were performed by means of Design-Expert 10 and SciLab 6.1 software programs. Additionally, the desorption of MB dye from the spent SPEEK beads was investigated in various liquid phases (see Figure S3 in Supplementary Materials). The highest MB desorption efficiency of 14.10% was noticed in ethanol.

### 3.4. Molecular Docking Simulations

**Figure 9:**
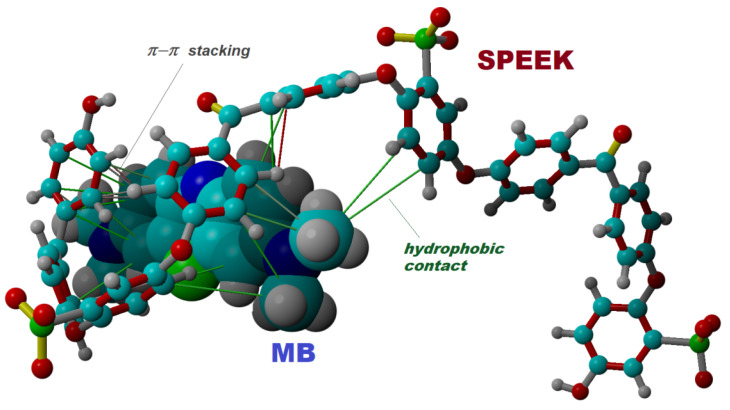
Molecular docking outcome: best pose of the docked complex showing the binding mode and interactions between SPEEK oligomer (receptor) and MB molecule (ligand).

To reveal some aspects of the adsorption mechanism at the molecular level, we investigated the interaction between the SPEEK oligomer and MB dye by using the molecular docking simulation. Molecular docking is a computational technique for predicting the binding mode and orientations of ligands (e.g., organic molecules) within receptors (e.g., oligomers, macromolecules, or supramolecular assemblies). In this computational study, the SPEEK trimer (in its anionic form) was modeled as the receptor, while the MB molecule in its cationic form was modeled as the ligand. The docking simulations were performed by means of the AutoDock-VINA algorithm [36] implemented in YASARA-Structure software (v.20.8.23) [37]. YASARA-Structure (from www.yasara.org) is an interactive real-time molecular modeling program sustained by a powerful visualization algorithm. In a typical simulation procedure, different poses (sterically allowed) of ligand conformation are probed, in the vicinity of the receptor. Hence, the possible conformations of docked complexes of type ligand-receptor are assessed. Then, a more energetically favorable docked pose is established (i.e., the best conformation of the docked complex). The docking computations were done at the level of molecular mechanics theory by adopting the YASARA force field, which relies on knowledge-based potentials for high accuracy. Such an approach enables the assessment of the total energy of the molecular system that can be expressed as a sum of individual contributions: bonds, angles, dihedrals, planarity, Van-der-Waals, and electrostatic terms. Assigning the force field parameters for the receptor and ligand was done automatically (using a so-called “AutoSMILES” algorithm) by YASARA-Structure. A Dell Precision workstation T7910 was used for all computer-aided simulations. Results of the molecular docking simulation are illustrated in Figure 9, where the best docking pose between SPEEK and MB is shown. For this docked complex (SPEEK@MB) the best energy score (affinity) was found to be −4.13 kcal/mol and the dissociation constant was equal to 0.939 mM. In addition, the interaction energy between receptor and ligand was calculated at the level of the YASARA force field [38] and found to be equal to ΔE = −31.79 kcal/mol for the studied system SPEEK/MB. From this value, −16.21 kcal/mol was attributed to Van-der-Waals forces, and −15.58 kcal/mol was attributed to Coulomb (or electrostatic) forces. Likewise, the molecular simulations revealed that the docked complex (SPEEK@MB) was stabilized by hydrophobic and π − π stacking interactions (Figure 9), which might be associated with the chemisorption mechanism suggested by the D–R isotherm experimental results. The chemisorption mechanism might also be responsible for the low desorption efficiency (0.04–14.10%), as the desorption study revealed for different types of eluents (see Figure S3 in Supplementary Materials).

## 4. Conclusions

In summary, we demonstrated a new approach to SPEEK preparation in a fast way by sulfonation of PEEK polymer at 60 °C using ultrasound.

The produced SPEEK in the form of polymer beads (2−4 mm) was characterized by advanced instrumental methods (SEM, EDAX, ^1^H-NMR, FTIR, and TG/DTG). As disclosed by the SEM analysis, the SPEEK beads were porous with pore sizes ranging from 1.13 μm to 151.44 μm that obey a log-normal distribution, indicating an average value of 44.31 μm. The degree of sulfonation (DS%) of SPEEK was found by ^1^H-NMR analysis to be equal to 70.3%, in acceptable agreement with DS (67 ± 2%) determined by the reverse titration method.

In adsorption assays, SPEEK beads were evaluated for the removal of MB cationic dye (organic pollutant) from synthetic wastewaters. The adsorption kinetics indicated the best-fitting models of adsorption kinetics, namely, the PnO model (pseudo-n-order) of order n = 1.18, followed by MOE (mix 1,2-order) and PFO (pseudo-first-order).

According to equilibrium studies (isotherms), the observed maximum adsorption capacities (q_e_) were equal to 217 mg/g, 119 mg/g, and 68 mg/g, for temperatures of 323 K, 313 K, and 300 K, respectively. The thermodynamic calculations revealed a positive enthalpy (ΔH_ad_ = +11.81 kJ/mol), indicating an endothermic effect of the adsorption process. The free energy of sorption, as determined from Dubinin–Radushkevich isotherms, ranged into the interval 22.21–35.03 kJ/mol, suggesting a chemisorption mechanism.

The influence of initial MB concentration (C_0_) and solution pH on the process response (color removal efficiency, Y %) was investigated by adopting the design of experiments, empirical modeling, and process optimization. The model-based optimization indicated the optimal conditions of separation were C_0_ = 89 mg/L and pH 2.8. Under these optimal conditions, the maximal color removal efficiency was found to be 99.14%, the highest value observed in the frame of this study. Regarding the desorption process, the most relevant MB desorption efficiency of 14.1% (from spent adsorbent) was observed in ethanol.

Finally, molecular docking simulations were done by a computer-assisted technique to reveal the intermolecular interactions between the SPEEK oligomer (receptor) and MB dye (ligand). The estimated interaction energy for attraction between ligand and receptor was equal to −31.79 kcal/mol, from which a contribution of 51% was attributed to VdW forces and 49% to Coulomb (electrostatic) forces. Moreover, the molecular docking pointed out that the receptor-ligand complex (SPEEK@MB) was stabilized by hydrophobic and π − π stacking interactions, which might be associated with the chemisorption mechanism as suggested by the evidence from the Dubinin–Radushkevich (D–R) isotherm.

## Figures and Tables

**Table 1:** Kinetic models and parameters for MB dye adsorption onto SPEEK beads, experimental conditions: T = 300 K, SD = 2 g/L, C_0_ = 100 mg/L, and pH 6.0 ± 0.2.

Model Abbreviation ^1^	Kinetic Model (Differential Equation)	Kinetic Model (Non-Linear Equation)	Kinetic Parameters
PFO	$\frac{{\mathrm{dq}}_{t}}{\mathrm{dt}}=k_{1}\left(q_{e}-q_{t}\right)$	$q_{t}=q_{e}\left(1-e^{-\mathrm{kt}}\right)$	q_e_ = 52.417 (mg/g) k_1_ = 1.076 × 10^−1^ χ^2^ = 0.2841
PSO	$\frac{{\mathrm{dq}}_{t}}{\mathrm{dt}}=k_{2}{\left(q_{e}-q_{t}\right)}^{2}$	$q_{t}=\frac{k_{2}q_{e}^{2}t}{1+k_{2}q_{e}t}$	q_e_ = 56.355 (mg/g) k_2_ = 2.772 × 10^−3^ χ^2^ = 1.072
PnO	$\frac{{\mathrm{dq}}_{t}}{\mathrm{dt}}=k_{n}{\left(q_{e}-q_{t}\right)}^{n}$	$q_{t}=q_{e}-{\left[\left(n-1\right)k_{n}t+q_{e}^{\left(1-n\right)}\right]}^{\frac{1}{1-n}}$	q_e_ = 52.806 (mg/g) k_n_ = 5.766 × 10^−2^ n = 1.179 χ^2^ = 0.0928
MOE	$\frac{{\mathrm{dq}}_{t}}{\mathrm{dt}}=\underset{i}{\sum }K_{i}{\left(q_{e}-q_{t}\right)}^{i}$	$q_{t}=q_{e}\frac{1-e^{\left(-K_{1}t\right)}}{1-\frac{K_{2}q_{e}}{K_{1}+K_{2}q_{e}}e^{\left(-K_{1}t\right)}}$	q_e_ = 52.701 (mg/g) K_1_ = 8.114 × 10^−2^ K_2_ = 7.801 × 10^−4^ χ^2^ = 0.0672
ID	$\frac{{\mathrm{dq}}_{t}}{\mathrm{dt}}=\frac{k_{d}}{2\sqrt{t}}$	$q_{t}=k_{d}\sqrt{t}+J$	k_d_ = 3.0452 J = 18.318 χ^2^ = 25.229

^1^ PFO—pseudo-first order kinetics; PSO—pseudo-second order kinetics; PnO—pseudo-n-order kinetics; MOE—mix 1,2-order kinetics; ID—intra-particle diffusion kinetics.

**Table 2:** Isotherm models and parameters for MB dye adsorption onto polymeric beads SPEEK, experimental conditions: SD = 2 g/L, t = 180 min, and pH 6.0 ± 0.2.

Isotherm Model		Parameters for the System SPEEK/MB		
	Isotherm Model Equation	T = 300 K (27 °C)	T = 313 K (40 °C)	T = 323 K (50 °C)
Freundlich	$q_{e}=K_{F}C_{e}^{1/n_{F}}$	K_F_ = 36.103 n_F_ = 8.672 χ^2^ = 1.3321	K_F_ = 86.932 n_F_ = 17.936 χ^2^ = 0.5512	K_F_ = 152.95 n_F_ = 12.529 χ^2^ = 8.3731
Langmuir	$q_{e}=\frac{q_{m}K_{L}C_{e}}{1+K_{L}C_{e}}$	q_m_ = 66.32 (mg/g) K_L_ = 2.8807 (L/mg) χ^2^ = 0.6536	q_m_ = 112.18 (mg/g) K_L_ = 3.9392 (L/mg) χ^2^ = 3.4076	q_m_ = 209.12 (mg/g) K_L_ = 3.9805 (L/mg) χ^2^ = 12.069
Redlich-Peterson (R-P)	$q_{e}=\frac{{\mathrm{AC}}_{e}}{1+B_{e}^{g}}$	A = 271.49 (L/g) B = 5.4143 (L/mg) g = 0.9461 χ^2^ = 0.0256	A = 441.89 (L/g) B = 3.9392 (L/mg) g = 1 χ^2^ = 3.4077	A = 436.50 (L/g) B = 2.1538 (L/mg) g = 0.9874 χ^2^ = 1.8879
Sips	$q_{e}=\frac{q_{s}{\mathrm{bC}}_{e}^{1/n}}{1+{\mathrm{bC}}_{e}^{1/n}}$	q_S_ = 76.446 (mg/g) b = 0.9315 n_S_ = 2.6375 χ^2^ = 0.0181	q_S_ = 1176.73 (mg/g) b = 0.0797 n_S_ = 16.380 χ^2^ = 0.5595	q_S_ = 209.58 (mg/g) b = 1.6955 n_S_ = 0.7104 χ^2^ = 1.8318
Dubinin-Radushkevichi	$E_{S}=\frac{1}{\sqrt{2K_{D}}}$	K_D_ = 1.013 × 10^−3^ E_S_ = 22.21 (kJ mol^−1^) r^2^ = 0.9912	K_D_ = 4.075 × 10^−4^ E_S_ = 35.03 (kJ mol^−1^) r^2^ = 0.9152	K_D_ = 5.926 × 10^−4^ E_S_ = 29.04 (kJ mol^−1^) r^2^ = 0.8306

**Table 3:** Central composite experimental design of rotatable type used for studying and enhancement of the adsorption system (SPEEK/MB dye); fixed conditions for experimentations: sorbent dose SD = 2 g/L; contact time t = 180 min; and temperature T = 323 K (50 °C).

Run Trial	Initial Concentration of Pollutant, MB (mg/L)		pH of Synthetic Aqueous Solutions		Response: Color Removal Efficiency, Recorded after 180 min Contact Time
	Coded	Actual	Coded	Actual	
	x_1_	C0 (mg/L)	x_2_	pH	Y (%)
1	−1	100	−1	4.0	97.96
2	+1	400	−1	4.0	85.34
3	−1	100	+1	10.0	97.65
4	+1	400	+1	10.0	95.42
5	−1.414	38	0	7.0	95.31
6	+1.414	462	0	7.0	78.81
7	0	250	−1.414	2.8	99.12
8	0	250	+1.414	11.2	99.00
9	0	250	0	7.0	98.83
10	0	250	0	7.0	98.88
11	0	250	0	7.0	98.95

**Table 4:** ANOVA (statistical test) for the developed mathematical model Ŷ(x_1_,x_2_).

Source	DF ^(a)^	SS ^(b)^	MS ^(c)^	F-Value ^(d)^	*p*-Value ^(e)^	R_2_ ^(f)^	R_adj_^2 (g)^
Model	4	425.52	106.38	26.44	0.0006	0.946	0.910
Residual	6	24.14	4.02	-	-	-	-
Total	10	449.66	-	-	-	-	-

^(a)^ degree of freedom; ^(b)^ sum of squares; ^(c)^ mean square; ^(d)^ ratio between mean squares; ^(e)^ probability of randomness; ^(f)^ coefficient of determination; ^(g)^ adjusted coefficient of determination.

## Data Availability

Not applicable.

## Supplementary Materials

The following supporting information can be downloaded at: https://www.mdpi.com/article/10.3390/ma15217558/s1, Section S1: Reverse titration method used to determine the degree of sulfonation; Figure S1: Suggestive scheme of SPEEK synthesis by ultrasonic-assisted sulfonation of PEEK (polyether ether ketone); Figure S2: Adsorption kinetics of heavy metals using SPEEK polymeric beads as sorbent (293 K); Table S1: Kinetics parameters for the studied adsorption process (SPEEK/heavy metal ions); Table S2: Thermodynamics parameters for the studied adsorption process (SPEEK/MB dye); Figure S3: Desorption efficiency of MB dye from spent SPEEK in various liquid phases.
