# Supplementary material for: Ultrasonic-Assisted Rapid Preparation of Sulfonated Polyether Ether Ketone (PEEK) and Its Testing in Adsorption of Cationic Species from Aqueous Solutions
Source: Materials (Basel). 2022 Oct 27;15(21):7558. doi: 10.3390/ma15217558 (PMC9654382; doi:10.3390/ma15217558)
Supplement: Supplementary file 1 [file materials-15-07558-s001.zip › materials-1955808-supplementary-update.pdf]

materials MDPI

Supplementary Materials

Section S1: Reverse titration method used to determine the degree of sulfonation

The sulfonation degree of the SPEEK was determined by back titration. For this purpose, 0.5 grams of polymer (m_P) was washed with distilled water and dried in an oven at 90 °C for 2 hours. Then, the polymer was washed with a mixture of 1:1 water and acetone, after which the material was washed again with pure acetone only. After the polymer was dried, it was introduced into 250 mL (V_t) of sodium hydroxide solution with a concentration of 0.01 M. After 72 hours, an aliquot of 9 mL (V_{NaOH}) of the partially neutralized sodium hydroxide solution was titrated with 0.01 M hydrochloric acid (HCl) in presence of phenolphthalein. The volume of HCl used to neutralize 9 mL (V_{NaOH}) was noted as V_{HCl}. To determine the degree of sulfonation (DS), firstly we calculated the ion exchange capacity (IEC). The IEC was determined using the following equation:

$$\mathrm{IEC} = \frac{(V_{NaOH} - V_{HCl})}{m_p} \times \frac{V_t}{V_{NaOH}}, \quad \text{(S1)}$$

With the neutralization of the NaOH solution by SPEEK, we can say that an SPEEK-Na copolymer is formed. The copolymer is composed of PEEK-SO_3Na units and PEEK units, the degree of sulfonation can be described using the molar mass of PEEK-SO_3Na (390 daltons) and PEEK (288 daltons):

$$\mathrm{GS} = \frac{M_{PEEK-SO_3Na}}{M_{PEEK-SO_3Na} + M_{PEEK}}, \quad \text{(S2)}$$

The molar mass of PEEK-SO_3Na unit ($M_{PEEK-SO_3Na}$) for one gram of SPEEK is:

$$M_{PEEK-SO_3Na} = 0.001\mathrm{IEC}, \quad \text{(S3)}$$

The molar mass of PEEK unit PEEK (M_{PEEK}) for one gram of SPEEK is:

$$M_{PEEK} = \frac{1 - 0.001 \times \mathrm{IEC} \times M_{PEEK-SO_3Na}}{M_{PEEK}}, \quad \text{(S4)}$$

Substituting Equation S2 and S3 in Equation S4, we obtain the equation for the sulfonation degree:

$$\mathrm{GS} = \frac{288 \times \mathrm{IEC}}{1000 - 102 \times \mathrm{IEC}}, \quad \text{(S5)}$$

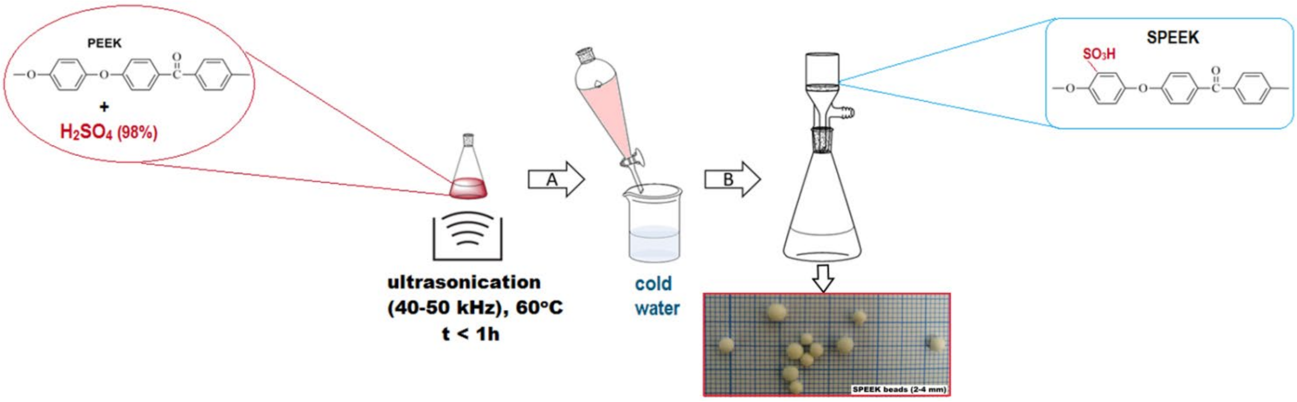

Figure S1. Suggestive scheme of SPEEK synthesis by ultrasonic-assisted sulfonation of PEEK (polyether ether ketone).

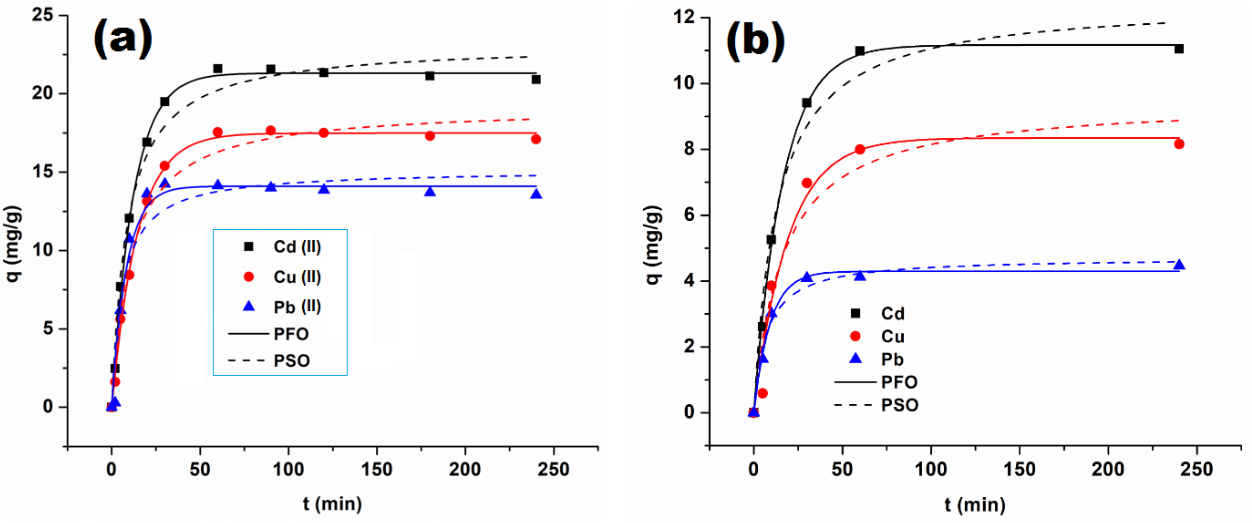

Figure S2. Adsorption kinetics of heavy metal cations (Cu^{2+}, Cd^{2+}, Pb^{2+}) onto the solid surface of SPEEK polymeric beads: (**a**) adsorption kinetics for one-component / individual systems (sorbent dose = 2 g/L; initial concentration of each metal ion C_0 = 50 mg/L; T = 300 K); (**b**) adsorption kinetics for competitive or multi-component system (sorbent dose = 2 g/L; initial concentration of each metal ion in the system was equal to 20 mg/L, summing up a total metal ions concentration of 60 mg/L; T = 300 K); solid lines designate predictions given by pseudo-first order (PFO) kinetic model, and dash lines represent predictions given by pseudo-second order (PSO) kinetic model.

The results on the individual and multi-component systems revealed a greater selectivity for the adsorption of Cd^{2+} ions, which were adsorbed in greater amounts compared to Cu^{2+} and then Pb^{2+} ions (Figure S2). Thus, for this case study, the adsorption capacities follow the order: $Cd^{2+} > Cu^{2+} > Pb^{2+}$ for the multi-component system (Figure S2b). This outcome suggests that the selectivity order is reversely correlated with the hydrated ionic radii of the cations (4.01 Å for Pb^{2+}, 4.19 Å for Cu^{2+}, and 4.26 Å for Cd^{2+}). It seems that the bulkiest hydrated cation Cd^{2+} might move more freely and quickly in aquatic media. Also, once the bulkiest hydrated cation Cd^{2+} was adsorbed on the solid surface they are hindering the access of the other cations to the active adsorption sites. In addition, the slower diffusion of Pb^{2+} might be also associated with its greater atomic weight compared to the other cations. The advantages of the produced adsorbent imply the form of beads, porous morphology, and selectivity for retention of cationic species due to functional groups (-SO_3^-).

Table S1. Kinetics parameters for the studied adsorption process (SPEEK / heavy metal ions) [1].

Kinetic parameters	PFO			PSO		
	Cd^{2+}	Cu^{2+}	Pb^{2+}	Cd^{2+}	Cu^{2+}	Pb^{2+}
**k**	0.08214	0.0693	0.11945	0.00492	0.00486	0.01094
**q_e**	21.32128	17.49069	14.10215	23.18836	19.19923	15.15105
**r^2**	0.99765	0.9974	0.96586	0.97959	0.97866	0.91579

[1] The concentrations of heavy metal ions (Cd^{2+}, Cu^{2+}, Pb^{2+}) were monitored by means of atomic absorption spectroscopy (AAS). The PFO kinetic model bestowed better predictions than the PSO model for the case of heavy metal ions adsorption on SPEEK.

Table S2. Thermodynamics parameters for the studied adsorption process (SPEEK / MB dye).

ΔG_{ad} (kJ/mol)	ΔH_{ad} (kJ/mol)	ΔS_{ad} (J/K.mol)
− 35.88 ± 1.76	+ 11.81 (endothermic)	152.84 ± 0.59

Thermodynamic parameters for the adsorption process were ascertained by using the following relationships [31]:

$$\Delta G_{ad} = -RT\ln(K_{ad}) \quad (S6)$$

$$\ln(K_{ad}) = \frac{\Delta S_{ad}}{R} - \frac{\Delta H_{ad}}{RT} \quad (S7)$$

where ΔG_{ad} (kJ/mol), ΔH_{ad} (kJ/mol), and ΔS_{ad} (J/(K·mol)) are the Gibbs free-energy, enthalpy and entropy of the adsorption, respectively; T is the absolute temperature (K); R stands for the universal gas constant (R=8.314 J/(K·mol)), and K_{ad} is the equilibrium constant of adsorption that was approximated to K_L parameter derived from Langmuir isotherm and converted to [L/mol] units (i.e., $K_{ad} \cong K_L$ [L/mol]), see for example [https://doi.org/10.1016/j.desal.2010.01.010] and [https://doi.org/10.2478/s11532-008-0019-2].

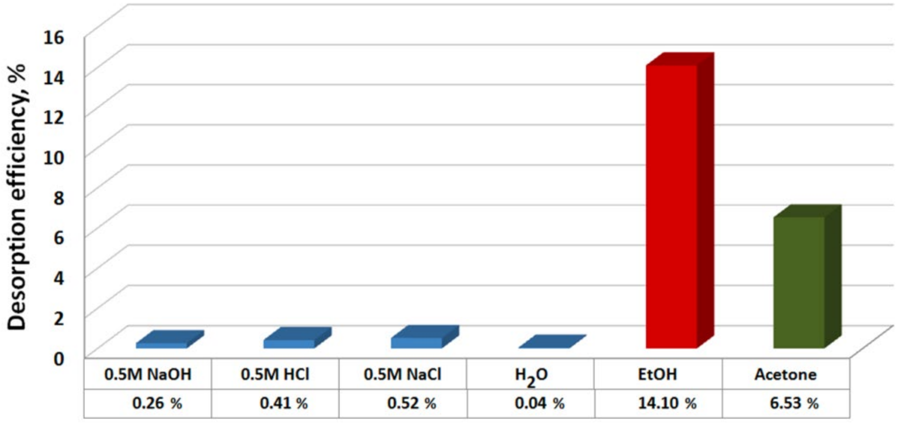

Figure S3. Desorption efficiency of MB dye from spent SPEEK in various liquid phases.
